# Mapping sex differences in the effects of protein and carbohydrates on lifespan and reproduction in *Drosophila melanogaster*: is measuring nutrient intake essential?

**DOI:** 10.1007/s10522-022-09953-2

**Published:** 2022-02-05

**Authors:** Matthew R. Carey, C. Ruth Archer, James Rapkin, Meaghan Castledine, Kim Jensen, Clarissa M. House, David J. Hosken, John Hunt

**Affiliations:** 1 Department of Metabolism, Digestion and Reproduction, Imperial College London, London, UK; 2 Centre for Ecology and Conservation, University of Exeter, Cornwall Campus, Cornwall, UK; 3 Institute of Evolutionary Ecology and Conservation Genomics, University of Ulm, Albert-Einstein Allee 11, 89069 Ulm, Germany; 4 Department of Animal Science - ANIS Nutrition, Aarhus University, Tjele, Denmark; 5 School of Science, Western Sydney University, Hawkesbury Campus, Richmond, NSW Australia

**Keywords:** Caloric restriction, *Drosophila melanogaster*, Geometric framework of nutrition, Longevity, Reproduction, CAFE assays

## Abstract

Understanding how diet affects reproduction and survival is a central aim in evolutionary biology. Although this relationship is likely to differ between the sexes, we lack data relating diet to male reproductive traits. One exception to this general pattern is *Drosophila melanogaster,* where male dietary intake was quantified using the **CA**pillary **FE**eder (CAFE) method. However, CAFE feeding reduces *D. melanogaster* survival and reproduction, so may distort diet-fitness outcomes. Here, we use the Geometric Framework of Nutrition to create nutrient landscapes that map sex-specific relationships between protein, carbohydrate, lifespan and reproduction in *D. melanogaster.* Rather than creating landscapes with consumption data, we map traits onto the nutrient composition of forty agar-based diets, generating broad coverage of nutrient space. We find that male and female lifespan was maximised on low protein, high carbohydrate blends (~ 1_P_:15.9_C_). This nutrient ratio also maximised male reproductive rates, but females required more protein to maximise daily fecundity (1_P_:1.22_C_). These results are consistent with CAFE assay outcomes. However, the approach employed here improved female fitness relative to CAFE assays, while effects of agar versus CAFE feeding on male fitness traits depended on the nutrient composition of experimental diets. We suggest that informative nutrient landscapes can be made without measuring individual nutrient intake and that in many cases, this may be preferable to using the CAFE approach. The most appropriate method will depend on the question and species being studied, but the approach adopted here has the advantage of creating nutritional landscapes when dietary intake is hard to quantify.

**Supplementary Information:**

The online version contains supplementary material available at 10.1007/s10522-022-09953-2.

## Introduction

In many species, dietary restriction (where individuals are fed less food but without causing malnutrition) extends lifespan but reduces fertility (Masoro 2005; Speakman and Mitchell 2011; Nakagawa et al. 2012; Simons et al. 2013). Longer lives in response to dietary restriction are taxonomically widespread—although most frequently observed in lab models (Nakagawa et al. 2012). Accordingly, dietary restriction has been described as a paradigm in aging biology (Anderson and Weindruch 2012) and understanding it is a major aim of aging research (Jensen et al. 2015; Moatt et al. 2020). A long-standing evolutionary explanation for greater lifespan under dietary restriction is that resources are reallocated away from reproduction towards somatic maintenance (Shanley and Kirkwood 2000). The rationale being that there is little gain to reproducing when food is limited and offspring survival prospects are poor. Instead, it is better to invest in surviving to reproduce when environmental conditions improve. This idea probably oversimplifies the relationship between nutrition, lifespan and reproduction (Adler and Bonduriansky 2014), and is unlikely to apply to all species e.g. long-lived species that reproduce over multiple seasons (Shanley and Kirkwood 2006). However, trade-offs involving reproduction may play a role in explaining why in some species, individuals live longer when they eat less (Moatt et al. 2020; Zanco et al. 2021).

The relationship between food, sex and death is not just about how much food individuals consume. The Geometric Framework of Nutrition has shown that both the amount of food consumed and its nutrient ratio interact to affect lifespan. For example a general trend in insects is that high protein, low carbohydrate intake reduces lifespan in both sexes (Lee et al. 2008; Maklakov et al. 2008; Fanson et al. 2009; Bruce et al. 2013; Harrison et al. 2014; Jensen et al. 2015; Malod et al. 2017; Rapkin et al. 2017). However, females typically require more protein for reproduction and therefore a single diet cannot maximise both lifespan and reproduction, resulting in a nutrient-based trade-off between these traits (Lee et al. 2008; Maklakov et al. 2008; Harrison et al. 2014; Archer et al. 2015; Rapkin et al. 2017). It is not clear if males face a similar trade-off because existing data suggest that in many insects, males can consume a single nutrient ratio to promote both lifespan and reproduction (Maklakov et al. 2008; Harrison et al. 2014; Jensen et al. 2015; Rapkin et al. 2017). However, our understanding of how nutrition affects male reproduction lags far behind our understanding of effects in females across all taxa, making it difficult to determine if this apparent sex-difference represents a general pattern. In particular, most research using the Geometric Framework of Nutrition has used insect models, and while accumulating research in other taxa has shown that nutrient blend as well as total nutrient intake affect reproductive effort and survival (e.g. mice Solon-Biet et al. 2015; e.g. sticklebacks Moatt et al. 2019), sex-differences in the relationship between nutrients and life-history are even less well understood outside of insects.

It has been suggested that failure to robustly characterise how diet affects male reproductive investment could have skewed our understanding of sex-differences in the relationship between nutrition and reproductive success (Moatt et al. 2016). While studies frequently test for effects of dietary restriction on lifespan in both sexes (see e.g. data used in meta-analysis by Nakagawa et al. 2012), far fewer studies test how dietary restriction affects reproduction (Moatt et al. 2016). Of those linking nutrition and reproduction, few assay males, and those that do seldom capture a significant proportion of male reproductive costs (Moatt et al. 2016). That is, to reproduce males must attract mates and fertilise their ova, often against a backdrop of intense male–male competition both before and after mating (Hosken and House 2011; Archer and Hosken 2021). Few dietary restriction studies assay how nutrition affects this full spectrum of male reproductive behaviours, and as a result, apparent sex-differences in the magnitude of dietary restriction impacts on reproduction may be an artefact of experimental design rather than a genuine biological signal (Moatt et al. 2016).

In the Geometric Framework of Nutrition literature, there have been tests of how nutrients interact to affect male reproductive traits including the number of offspring sired (Reddiex et al. 2013; Jensen et al. 2015), sexual signalling (Maklakov et al. 2008; South et al. 2011; Harrison et al. 2014; Archer et al. 2015; Bunning et al. 2016; Rapkin et al. 2017; Moatt et al. 2019) and sperm number and viability (Bunning et al. 2015; Morimoto and Wigby 2016). However, few studies have tested how nutrition affects lifespan and reproduction in both sexes concurrently (see however Lee et al. 2008; Harrison et al. 2014; Archer et al. 2015; Rapkin et al. 2017; Moatt et al. 2019). Once more, existing studies typically measure female reproduction directly (i.e. fecundity in mated females), but use proxy measures of male reproductive effort. For example in field crickets calling effort is frequently used as a measure of male reproduction (Archer and Hunt 2015). Such proxies only provide a snapshot of how diet affects male reproductive investment and do not assess copulation and ejaculation costs, which can represent a major portion of male reproductive effort (Martin and Hosken 2004; Brown et al. 2009). There are exceptions to this however, with studies showing that protein and carbohydrate affect both female fecundity and male competitive siring success in *Drosophila melanogaster* (Reddiex et al. 2013; Jensen et al. 2015). However, these *Drosophila* studies come with some caveats. For example, Reddiex et al. (2013) used yeast as a protein source; yeast contains more nutrients than just protein and so diets varying in their yeast content inevitably vary in more than just dietary protein. This makes it more complicated to determine which specific nutrients affected male phenotypes compared to work using fully chemically defined (i.e. holidic) diets. Additionally, Reddiex et al. (2013) only tested for effects on early-life reproduction and did not test for lifespan impacts. Finally, both Jensen et al. (2015) and Reddiex et al. (2013) used the CApillary FEeder (CAFE) method (Ja et al. 2007) to measure the nutrient intake of flies.

By providing liquid diets in a micro-capillary, the CAFE method enables the quantification of food consumption in small invertebrates such as *Drosophila* (Ja et al. 2007), and so its use can reveal how intake may vary with dietary dilution (e.g. detect compensatory feeding). Accurately quantifying nutrient intake is central to creating nutrient landscapes using the Geometric Framework of Nutrition to determine how each nutrient independently, and via interactions with other nutrients, affects phenotype (Simpson and Raubenheimer 1995, 2007, 2012). However, while the CAFE method enables accurate quantification of nutrient intake (Deshpande et al. 2014), this approach reduces lifespan and reproduction in *Drosophila* (Lee et al. 2008; Jensen et al. 2015). Worryingly, sex-differences in ethanol preferences not seen in CAFE assays emerge when solid foods are provided and intake measured using tracer methods (Park et al. 2018). If the CAFE approach (or similar methods e.g. feeding using pipette tips) can alter sex-differences in responses to a particular dietary treatment, it is possible that the widespread use of the CAFE approach in small invertebrates (e.g. Lee et al. 2008; Fanson et al. 2009; Reddiex et al. 2013; Jensen et al. 2015; Malod et al. 2017) has skewed our understanding of the sex-specific relationship between nutrition and phenotype.

With this in mind, we tested how consumption of protein and carbohydrates affected lifespan and reproduction in *D. melanogaster*, using broad measures that capture most of the costs of female and male reproduction. Additionally, we tested whether it was possible to create informative nutrient landscapes without collecting consumption data. That is, can we avoid the costs of CAFE (reduced fitness) without losing the insights offered by the accurate quantification of food consumption? Here, we dispense with creating nutrient landscapes using individual consumption data, and plot trait values onto the protein and carbohydrate content of experimental diets. To generate broad coverage of the nutrient space, we utilise more diets than is common in Geometric Framework studies: 800 flies of each sex were fed one of 40 fully chemically defined (i.e. holidic) diets that varied in their ratio and amount of protein and carbohydrate.

## Methods and materials

### Fly stock and maintenance

Dahomey *Drosophila melanogaster* stocks (supplied by Nick Priest, University of Bath) and Krüppel mutation stocks (Bloomington Stock Centre, received September 2015) were maintained in each of two large population cages (1m^3^) with overlapping generations at 25 °C under a 12:12 light:dark cycle. Stocks were maintained at ~ 2000 individuals allowed to mate freely, and fed ‘Jazz mix’ diet (Fisher Scientific, Loughborough, UK), provided in wide-neck 1000 ml jars. Stock cultures were maintained using this protocol for *ca.* 9 months prior to use. Experimental animals were cultivated using small vials (25 mm × 95 mm). To collect experimental animals, multiple small vials were put into each of the two population cages for a maximum of 6 h. These vials were then incubated while flies developed. Virgin flies of both sexes were collected from both of the Dahomey and Krüppel population cages within 4 h of eclosion, and then allocated at random to one of 40 dietary treatments.

### Artificial diets

In total, 40 artificial, holidic (i.e. fully chemically defined) diets that varied in both protein (P) to carbohydrate (C) ratio (i.e. P:C) and total nutritional content (i.e. P + C), were created using the established protocol outlined in Simpson and Abisgold (1985). Diets varied along 10 different P:C ratios (3:1, 2:1, 1.5:1, 1:1, 1:1.5, 1:2, 1:3, 1:5, 1:8, 1:16) and were diluted with indigestible cellulose such that they contained 12, 36, 60 or 80% total nutrition (i.e. P + C) (for detail please see Fig. S1 and Table S1). Powdered forms of each diet were created using the methods outlined in South et al. (2011) and then combined with water, agar and Nipagin (ratio—10:10:1:0.1 diet, water, agar and Nipagin). To achieve this, agar was added to water, which was boiled and then left to cool to < 60 °C, after which Nipagin and each powered diet was added. Black food colouring was added to foods to increase the contrast between eggs and food to make egg counting easier. These agar-based diets were provided in ‘vial caps’ (1.6 cm diameter, 1.6 cm deep) that could be securely fitted to the vials in which experimental individuals were housed. Caps were changed every 5 days both before and after mating. The experimental diets provided food, moisture and oviposition substrate for experimental females.

### Experimental protocol

A total of 20 virgin flies of each sex were assigned to each of 40 artificial diets at random (*N* = 1600) on the day of hatching and housed individually in a vial containing experimental diet and kept under the same light:dark regime as stock populations. Lifespan and reproduction were measured in the same experimental animals: lifespan by monitoring survival daily from adult eclosion and reproductive effort by counting eggs (females) or offspring sired in a competitive mating assay (males). We commenced measuring female fecundity at day five post hatching, by pairing each focal female at random with a 5-day-old virgin male mating partner for 12-h. Mating partners were collected and housed in the same manner as experimental individuals until mating. This mating regime was continued for the duration of an experimental individual’s lifetime (i.e. females were paired with mates every 5 days, for 12-h, beginning at 18:00–19:00 shortly before lights went out in experimental incubators) and each time a new 5-day-old mating partner was used. This regime was designed to ensure that females did not suffer reduced fecundity due to sperm limitation, or reduced longevity due to the direct costs of mating and male harassment (rationale in Taylor et al. 2008). All experimental flies had their food caps changed 24 h prior to mating. The food caps were also changed 6 h after mating partner removal, at which time female egg production was counted under a binocular microscope. This regime is summarised in Fig. S2 in the online supplement.

Male reproductive effort, also measured every 5 days, was quantified using similar methods to those outlined in Jensen et al. (2015). In brief, we counted the number offspring produced by a focal male when in competition with a 5-day-old virgin male with the Krüppel dominant eye mutation with a wild-type female. This allowed for offspring paternity to be easily assigned and provided us with a biologically relevant measure of male fitness that we could compare to previous work (i.e. siring success). After the 12-h mating period, the competing Krüppel male and the wild-type tester female were removed, and the female established on 7 ml of standard ‘Jazz mix’ diet for 14 days. On day 14, vials were frozen and wild-type and Krüppel counted. This regime is summarised in Fig. S3 in the online supplement.

Reproductive effort was assessed every 5 days across an individual’s lifetime. Total reproductive effort equates to the sum of these measures (females—total eggs laid, males—total offspring sired) and daily reproductive effort is the average reproductive output per mating opportunity (females—the average number of eggs produced per mating opportunity, males—the average number of offspring sired per mating opportunity). Daily reproductive effort was calculated as the average of each count because each reproductive assay captured around 1 days’ worth of reproductive activity i.e. up to 18 h of egg laying for females, and one competitive mating trial for males. Flies that died before their first mating or escaped during the experiment were replaced, resulting in a sample size of 1600 individuals (800 males and 800 females).

### Statistical analysis

A multivariate response-surface approach outlined in South et al. (2011) was used to estimate the linear and nonlinear (i.e. quadratic and correlational) effects of protein (hereafter, P) and carbohydrate (C) intake on lifespan, daily reproductive effort and total reproductive effort within each sex. To visualise the multivariate nutritional landscapes for each trait, non-parametric thin-plate splines were constructed in R using the *Tps* function in the Fields package (Nychka et al. 2015) of R (R Core Development Team, version 3.1.2, Vienna, Austria, www.r-project.org). The locations of the nutritional optima and their 95% confidence regions (CRs) were optimised using the *OptRegionTps* function in the ‘OptimaRegion’ package (del Castillo et al. 2020). Full details of this approach is provided in Rapkin et al. (2018).

A sequential model-building approach (Draper and John 1988) was used to determine whether the linear and nonlinear (quadratic and correlational) effects of nutrient intake differed across our response variables (lifespan, daily reproductive effort and total reproductive effort). Full details of this approach are outlined in Text S1. The sequential model building approach can quantify differences in linear and nonlinear gradients for different response variables but cannot quantify the direction of this difference in nutritional space (Rapkin et al. 2015; Bunning et al. 2016). It is possible for response variables to show differences in the magnitude of linear and nonlinear gradients, but at the same time occupy a similar location in nutritional space. Two additional measures were therefore calculated to quantify any difference in the location of nutritional optima. First, the angle (${\varvec{\theta}}$) and 95% confidence interval (CI) between nutritional vectors for the two response variables of interest were calculated using the procedure outlined in Bunning et al. (2015) (Fig. S4). Second, the divergence between the global nutritional maxima (calculated from 95% CR of the nutritional landscape) was estimated using the Euclidean distance (***d***) and corresponding 95% CIs using the *CRcompare* function in the ‘OptimaRegion’ package in R (Fig. S4). See Rapkin et al. (2018) for a full overview and justification of this analysis.

## Results

### Nutrient effects on lifespan and reproduction in the sexes

**Fig. 1:**
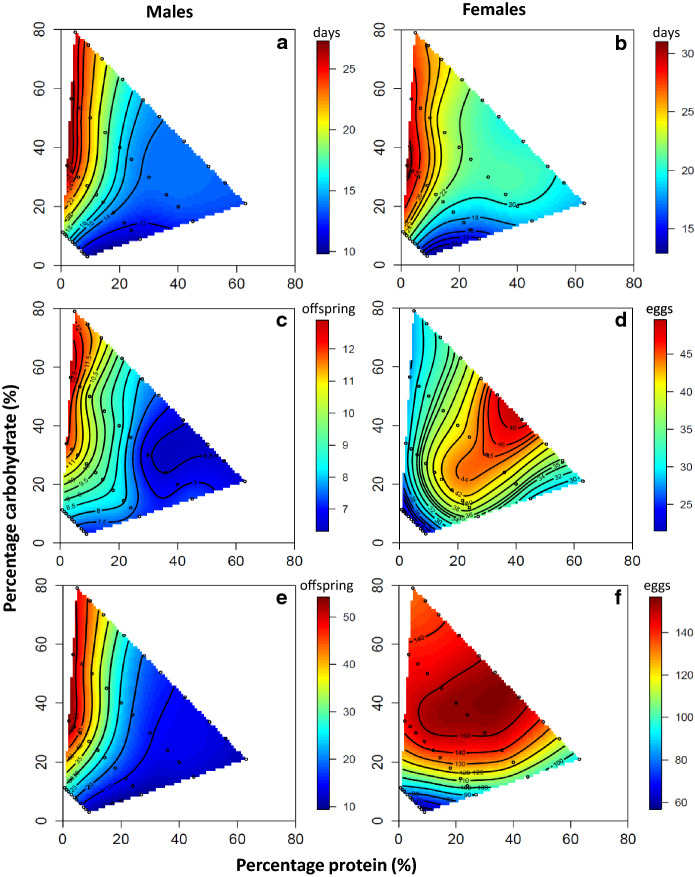
Nutrient landscapes relating dietary protein and carbohydrate to sex-specific lifespan (LS) and reproductive traits. Nonparametric thin-plate spline contour visualizations of the responses surfaces describing the effects of protein and carbohydrate intake on **a** male LS, **b** female LS, **c** male offspring production rate, **d** female egg production rate, **e** total offspring production in males, and **f** total egg production in females in *Drosophila melanogaster*

**Fig. 2:**
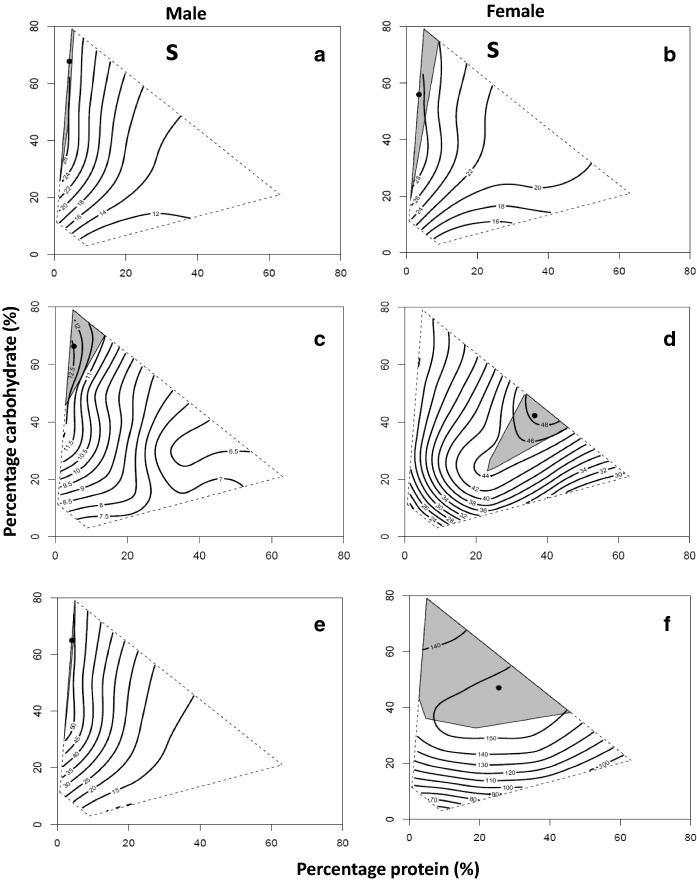
Confidence regions surrounding dietary optima. Nutritional optima are given as X,Y coordinates: **a** male lifespan = 4.25,67.70, **b** female lifespan = 3.53,56.07, **c** male daily reproductive effort = 5.19, 66.32, **d** female daily reproductive effort = 36.50, 42.22, **e** male total reproductive effort = 4.08,64.96, **f** female total reproductive effort = 25.43,47.10. Nutritional optima are shown as black points and the grey regions surrounding these are 95% confidence intervals

In both sexes, increased intake of dietary protein reduced lifespan but dietary carbohydrates increased it (Table 1, Fig. 1a, b). Both nutrients also had significant quadratic effects on lifespan in the sexes. For dietary carbohydrates the quadratic coefficient was negative for both sexes, meaning that there was a peak in lifespan on the nutritional landscape for this nutrient (Table 1; Fig. 1a, b). For dietary protein, the quadratic coefficient was positive for both sexes, reflecting a minimum lifespan at medium to high values of this nutrient on the nutritional landscape (due to the optima in lifespan being at low levels of dietary protein; Fig. 1a, b). There was also a significant negative correlational effect between dietary protein and carbohydrate on lifespan in both sexes, further highlighting the increase in lifespan on diets low in protein and high in carbohydrate (Table 1, Fig. 1a, b). Indeed, formal analysis revealed that lifespan was maximised at a P:C ratio of 1_P_:15.93_C_ in males (Fig. 2a) and 1_P_:15.88_C_ in females (Fig. 2b).

**Table 1:** The linear (P) and carbohydrate (C), quadratic (P × P; C × C) and correlational (P × C) effects of protein (P) and carbohydrates (C) on lifespan (LS), daily reproductive effort (DRE) and total reproductive effort (TRE) for male and female *Drosophila melanogaster*

Response variables	Linear effects		Nonlinear effects		
	P	C	P × P	C × C	P × C
(A) Males					
* LS*					
Coefficient ± SE	− 0.31 ± 0.03	0.38 ± 0.03	0.27 ± 0.03	− 0.31 ± 0.03	− 0.32 ± 0.05
* t*_799_	9.94	12.14	9.99	10.20	7.00
* P* value	0.0001	0.0001	0.0001	0.0001	0.0001
* DRE*					
Coefficient ± SE	− 0.15 ± 0.04	0.15 ± 0.04	0.04 ± 0.03	− 0.08 ± 0.04	− 0.17 ± 0.06
* t*_799_	4.30	4.20	1.29	2.17	2.91
* P* value	0.0001	0.0001	0.19	0.03	0.004
* TRE*					
Coefficient ± SE	− 0.30 ± 0.03	0.30 ± 0.03	0.20 ± 0.03	− 0.25 ± 0.03	− 0.30 ± 0.05
* t*_799_	9.34	9.38	6.68	7.62	5.94
* P* value	0.0001	0.0001	0.0001	0.0001	0.0001
(B) Females					
* LS*					
Coefficient ± SE	− 0.17 ± 0.03	0.25 ± 0.03	0.18 ± 0.03	− 0.26 ± 0.04	− 0.12 ± 0.05
* t*_799_	5.13	7.39	5.55	7.45	2.15
* P* value	0.0001	0.0001	0.0001	0.0001	0.032
* DRE*					
Coefficient ± SE	0.27 ± 0.03	0.17 ± 0.03	− 0.27 ± 0.03	− 0.21 ± 0.03	0.04 ± 0.05
* t*_799_	7.92	4.95	8.56	6.20	0.79
* P* value	0.0001	0.0001	0.0001	0.0001	0.43
* TRE*					
Coefficient ± SE	0.09 ± 0.03	0.33 ± 0.03	− 0.09 ± 0.03	− 0.35 ± 0.03	− 0.07 ± 0.05
* t*_799_	2.63	9.81	2.80	10.20	1.32
* P* value	0.009	0.0001	0.005	0.0001	0.19

*SE* standard error and *t799* the test of the coefficient with 799 degrees of freedom

Statistical comparison of nutritional landscapes for lifespan across the sexes revealed significant sex differences in the linear, quadratic and correlational effects of dietary protein and carbohydrates (Table 2). The significant sex difference in linear effects of dietary protein and carbohydrates occurred because lifespan decreased more steeply with increased dietary protein and increased more steeply with carbohydrates in males than in females (Table 2). The significant sex difference in quadratic effects was driven exclusively by the protein content of the diet and occurred because the coefficient was more positive in males than in females (Table 2). The significant sex difference in the correlational effects existed because the coefficient was more negative in males than in females (Table 2). However, despite the nutrient effects on lifespan being consistently greater in males than in females, the small angle between the linear nutritional vectors (***θ* = **12.50°, 95% CIs: 3.27°, 20.76°) and the small Euclidean distance between the optima (***d*** = 18.64, 95% CIs: 15.67, 21.06), indicates that the optima for lifespan occupied similar regions on the nutritional landscape for the sexes (Figs. 1a, b, 2a, b).

**Table 2:** Sequential model building analysis that contrasts the linear and nonlinear effects of protein (P) and carbohydrate (C) on lifespan (LS), daily reproductive effort (DRE) and total reproductive effort (TRE), both between the sexes, and between traits within the sexes

	*SS*_R_	*SS*_C_	*DF*_1_	*DF*_2_	*F*	*P*
Males vs. females						
* LS*						
Linear	1356.44	1343.05	2	1594	7.95	0.0004^A^
Quadratic	1169.55	1164.35	2	1590	3.55	0.03^B^
Correlational	1137.26	1131.30	1	1588	8.37	0.004
* DRE*						
Linear	1552.56	1483.24	2	1594	37.25	0.0001^C^
Quadratic	1433.03	1375.95	2	1590	32.98	0.0001^D^
Correlational	1373.69	1367.47	1	1588	7.22	0.007
* TRE*						
Linear	1425.72	1365.03	2	1594	35.44	0.0001^E^
Quadratic	1254.97	1207.66	2	1590	31.14	0.0001^F^
Correlational	1188.58	1181.13	1	1588	10.02	0.002
Male						
* LS vs. DRE*						
Linear	1411.59	1380.95	2	1594	17.68	0.0001^G^
Quadratic	1297.67	1262.59	2	1590	22.09	0.0001^H^
Correlational	1228.69	1225.36	1	1588	4.31	0.038
* LS vs. TRE*						
Linear	1276.97	1274.67	2	1594	1.43	0.24
Quadratic	1097.12	1094.11	2	1590	2.19	0.11
Correlational	1039.76	1039.68	1	1588	0.12	0.74
* DRE vs. TRE*						
Linear	1442.96	1424.74	2	1594	10.19	0.0001^I^
Quadratic	1369.81	1352.18	2	1590	10.37	0.0001^J^
Correlational	1321.39	1318.99	1	1588	2.89	0.09
Female						
* LS vs. DRE*						
Linear	1524.23	1445.34	2	1594	43.50	0.0001^K^
Quadratic	1364.34	1277.71	2	1590	53.90	0.0001^L^
Correlational	1276.93	1273.41	1	1588	4.39	0.04
* LS vs. TRE*						
Linear	1463.56	1433.41	2	1594	16.76	0.0001^M^
Quadratic	1312.80	1277.90	2	1590	21.71	0.0001^N^
Correlational	1273.06	1272.75	1	1588	0.40	0.53
* DRE vs. TRE*						
Linear	1445.75	1423.52	2	1594	12.45	0.0001^O^
Quadratic	1249.83	1231.44	2	1590	11.87	0.0001^P^
Correlational	1231.33	1229.61	1	1588	2.23	0.14

Univariate test: ^A^P: *F*_1,1594_ = 8.73, *P* = 0.003, C: *F*_1,1594_ = 7.74, *P* = 0.005; ^B^P × P: *F*_1,1590_ = 7.08, *P* = 0.008, C × C: *F*_1,1590_ = 0.20, *P* = 0.66; ^C^P: *F*_1,1594_ = 73.96, *P* = 0.0001, C: *F*_1,1594_ = 0.18, *P* = 0.67; ^D^P × P: *F*_1,1590_ = 51.60, *P* = 0.0001, C × C: *F*_1,1590_ = 9.10, *P* = 0.003; ^E^P: *F*_1,1594_ = 70.16, *P* = 0.0001, C: *F*_1,1594_ = 0.29, *P* = 0.59; ^F^P × P: *F*_1,1590_ = 50.91, *P* = 0.0001, C × C: *F*_1,1590_ = 6.80, *P* = 0.009; ^G^P: *F*_1,1594_ = 11.79, *P* = 0.001, C: *F*_1,1594_ = 24.78, *P* = 0.0001; ^H^P × P: *F*_1,1590_ = 30.19, *P* = 0.0001, C × C: *F*_1,1590_ = 18.45, *P* = 0.0001; ^I^P: *F*_1,1594_ = 10.23, *P* = 0.001, C: *F*_1,1594_ = 10.90, *P* = 0.001; ^J^P × P: *F*_1,1590_ = 13.39, *P* = 0.0001, C × C: *F*_1,1590_ = 9.48, *P* = 0.002; ^K^P: *F*_1,1594_ = 85.00, *P* = 0.0001, C: *F*_1,1594_ = 3.08, *P* = 0.08; ^L^P × P: *F*_1,1590_ = 107.66, *P* = 0.0001, C × C: *F*_1,1590_ = 0.51,* P* = 0.47; ^M^P: *F*_1,1594_ = 30.19, *P* = 0.0001, C: *F*_1,1594_ = 2.63, *P* = 0.11; ^N^P × P: *F*_1,1590_ = 37.14, *P* = 0.0001, C × C: *F*_1,1590_ = 3.44, *P* = 0.06; ^O^P: *F*_1,1594_ = 14.26, *P* = 0.0001, C: *F*_1,1594_ = 11.53, *P* = 0.001; ^P^P × P: *F*_1,1590_ = 19.02, *P* = 0.0001, C × C: *F*_1,1590_ = 6.86, *P* = 0.009

Male daily and total reproductive effort increased with dietary carbohydrates and decreased with increased dietary protein (Table 1a; Fig. 1c, e). As for male lifespan, there was a significant positive quadratic effect of dietary protein for total reproductive effort (but this was not significant for daily reproductive effort) and a significant negative quadratic effect of dietary carbohydrates on both traits (Table 1a; Fig. 1c, e). Likewise, there was also a significant negative correlational effect between dietary protein and carbohydrates on daily and total reproductive effort (Table 1a; Fig. 1c, e). Formal analysis revealed that daily reproductive effort was maximised at a P:C ratio of 1_P_:12.78_C_ (Fig. 2c) and total reproductive effort at 1_P_:15.92_C_ (Fig. 2e).

In females, daily reproductive effort and total reproductive effort both increased with dietary protein and dietary carbohydrates (Table 1b, Fig. 1d, f). There were also significant negative quadratic coefficients for dietary protein and carbohydrates indicating a well-defined peak for daily (Fig. 1d) and total reproductive effort (Fig. 1f). The correlational effect between dietary protein and carbohydrates, however, was not significant for either daily or total reproductive effort (Table 1b). Formal analysis revealed that daily reproductive effort was maximised at a P:C ratio of 1_P_:1.16_C_ (Fig. 2d) and total reproductive effort at 1_P_:1.85_C_ in females (Fig. 2f).

Statistical comparison of the nutritional landscapes for daily and total reproductive effort across the sexes revealed significant sex differences in the linear, quadratic and correlational effects of dietary protein and carbohydrates (Table 2). For both daily reproductive effort and total reproductive effort, the significant sex difference in the linear effects of nutrients was driven exclusively by dietary protein and the fact that daily reproductive effort and total reproductive effort increased with dietary protein in females but decreased with this nutrient in males (Table 2). The significant sex difference in the quadratic effects of nutrients was due to a positive quadratic coefficient for dietary protein in males but a negative coefficient in females and the quadratic coefficient for dietary carbohydrates being more negative for females than males (indicative of a more pronounced peak) (Table 2). The significant sex difference in the correlational effects existed because the coefficient was negative in males but not significant in females (Table 2). These pronounced sex differences in the magnitude and sign of nutritional effects resulted in the optima for daily and total reproductive effort being located in different regions on the nutritional landscape for males (Figs. 1c, e, 2c, e) and females (Figs. 1d, f, 2d, f), as evidenced by the large angles between the linear nutritional vectors (daily reproductive effort: ***θ*** = 33.26°, 95% CIs 22.27°, 44.31°; total reproductive effort: ***θ*** = 44.35°, 95% CIs 35.58°, 53.73°) and the large Euclidean distance between the nutritional optima (daily reproductive effort: ***d*** = 43.01, 95% CI 42.12, 43.63; total reproductive effort: ***d*** = 34.53, 95% CIs 33.30, 35.14) for the sexes.

### The trade-off between lifespan and reproduction within the sexes

Sequential model testing revealed that there were significant differences in the linear and nonlinear effects of nutrients on lifespan and daily reproductive effort in males (Table 2). The significant difference in the linear effects occurred because the negative impacts of dietary protein and the positive effects of dietary carbohydrates were stronger for lifespan than daily reproductive effort (Table 2). Likewise, the significant difference in quadratic effects was due to the quadratic coefficients for dietary protein being more positive, but the coefficient for dietary carbohydrates being more negative, for lifespan than daily reproductive effort (Table 2). The significant difference in the correlational effects was due to the correlational coefficient being more negative for lifespan than daily reproductive effort. In contrast, the linear and nonlinear effects of dietary protein and carbohydrates did not differ significantly between lifespan and total reproductive effort (Table 2). However, the linear and quadratic (but not the correlational) effects of these nutrients differed significantly between daily reproductive effort and total reproductive effort (Table 2). The difference in linear effects reflects that the negative effects of dietary protein and the positive effects of dietary carbohydrates were stronger for total reproductive effort than for daily reproductive effort (Table 2). The significant difference in quadratic effects was due to the quadratic coefficients for dietary protein being more positive, and the coefficient for dietary carbohydrates being more negative, for total reproductive effort than for daily reproductive effort (Table 2).

Collectively, the differences observed in our sequential models for males were all due to changes in the magnitude of nutrient effects on lifespan, daily reproductive effort and total reproductive effort, rather than changes in the sign or significance of these effects. This suggests that the optima for lifespan, daily reproductive effort and total reproductive effort in males are located in similar regions on the nutritional landscape (Figs. 1a, c, e, 2a, c, e). Accordingly, the angle between the linear nutritional vectors and the Euclidean distances between the nutritional optima for lifespan and daily reproductive effort (***θ* = **6.73°, 95% CIs 0.00°,15.52°, ***d*** = 12.81, 95% CIs 11.08, 14.35), lifespan and total reproductive effort (***θ* = **22.90°, 95% CIs 13.68°, 31.36°; ***d*** = 13.88, 95% CIs 12.26, 15.55) and daily reproductive effort and total reproductive effort (***θ* = **28.61°, 95% CIs 16.89°,40.88°; ***d*** = 16.00, 95% CIs 14.47, 17.67) were all modest. In particular, the small angle between the nutritional optima for lifespan and daily reproductive effort suggests that any nutrient-based trade-off between these traits is likely to be weak in males.

In females, there were significant differences in the linear, quadratic and the correlational effects of dietary protein and carbohydrates on lifespan and daily reproductive effort (Table 2). The significant difference in the linear effects occurred because daily reproductive effort increased with dietary protein but lifespan decreased with this nutrient (Table 2). The significant difference in the quadratic effects was due to the quadratic coefficient for dietary protein being positive for lifespan but negative for daily reproductive effort (Table 2). Similar differences in the linear and quadratic (but not correlational) effects of dietary protein and carbohydrates were found for lifespan and total reproductive effort (Table 2). Again, significant differences in the linear effects occurred because total reproductive effort increased with dietary protein but lifespan decreased with this nutrient. The significant differences in the quadratic effects were due to the quadratic coefficient for dietary protein being positive for lifespan but negative for total reproductive effort (Table 2). There were significant differences in the linear, quadratic but not the correlational effects of dietary protein and carbohydrates on daily reproductive effort and total reproductive effort (Table 2). Significant difference in linear effects occurred because there was a stronger positive effect of dietary protein on daily reproductive effort than on total reproductive effort, while the opposite pattern was true for dietary carbohydrates (Table 2). The significant difference in the quadratic effects was the result of the quadratic coefficient for dietary protein being more negative for daily reproductive effort than for total reproductive effort but again the opposite pattern was true for dietary carbohydrates (Table 2).

Collectively, the differences observed in our sequential models for females were due to changes in the sign and magnitude of nutrient effects on lifespan, daily reproductive effort and total reproductive effort, suggesting that optima for the traits are located in different regions of the nutritional landscape (Figs. 1b, d, f, 2b, d, f). In agreement with this view, both the angle between the linear nutritional vectors and the Euclidean distance between the nutritional optima for lifespan and daily reproductive effort (***θ*** = 26.48, 95% CIs 17.97°, 34.45°; ***d*** = 40.06 95% CIs 39.51, 40.58), were larger in females than males. This suggests that nutrient-based trade-offs between lifespan and daily reproductive effort are likely to be more substantial in females than males. However, the angles and distances between lifespan and total reproductive effort (***θ*** = 9.16, 95% CIs 0.36°, 16.88°;*** d*** = 32.96, 95% CIs 31.92, 33.96) and daily reproductive effort and total reproductive effort (***θ*** = 17.41, 95% CIs 9.16°, 25.20°; ***d*** = 14.48, 95% CIs 13.89, 15.13) were modest.

## Discussion

Understanding how diet affects lifespan, reproduction and the trade-off between these traits is a key aim in life-history biology and fundamental to aging research (Moatt et al. 2020; Regan et al. 2020). However, a lack of data testing how diet affects the full spectrum of male reproductive costs (Moatt et al. 2016) means that sex differences in the relationship between food, sex and death remain poorly understood (Moatt et al. 2020). The limited data available testing how diet affects survival and reproduction in both sexes concomitantly, largely come from *D. melanogaster.* These data are complicated because flies are typically fed via the CAFE method, which has clear and costly effects on overall fitness (Lee et al. 2008; Jensen et al. 2015). Here, we used agar-based diets to test how protein and carbohydrate affect survival and reproduction in both sexes, creating nutrient landscapes without measuring consumption. We find that longevity was greatest on high carbohydrate, low protein diets in both sexes. However, these nutrients had sex-specific effects on reproduction; male daily siring success was maximized on low protein, high carbohydrate diets but female daily fecundity was maximized in flies fed diets richer in protein. The general topography of the nutrient landscapes we created are similar to those created using the CAFE approach, but the absolute values for lifespan and reproduction we report were generally higher—particularly for females. While quantifying food intake is key to tackling many research questions, our results show that it is possible to create high resolution and informative nutritional landscapes, while avoiding the limitations of CAFE approach.

In agreement with a wealth of previous research, lifespan and reproduction depended on both the total amount and specific blend of nutrients that individuals consumed, rather than on energy alone (Mair et al. 2005; Lee et al. 2008; Maklakov et al. 2008; Fanson et al. 2009; Nakagawa et al. 2012; Bruce et al. 2013; Jensen et al. 2015; Le Couteur et al. 2016; Malod et al. 2017; Piper et al. 2017; Rapkin et al. 2017). In both sexes, lifespan was greatest on low protein, high carbohydrate diets (female: 1_P_:15.88_C_, male: 1_P_:15.93_C_). Why lifespan is often greatest in individuals consuming low protein diets is not fully understood. One possibility is that individuals fed high protein diets use protein for energy, and in doing so incur a metabolic cost. This metabolic penalty could be associated with transformations and waste production associated with protein deamination and prevention of toxicity (Anderson et al. 2020). What is clear is that this is a general trend, with increased lifespan on low protein diets being documented in insect species including Queensland fruit flies (*Bactrocera tryoni*) (Fanson et al. 2009; Fanson and Taylor 2012), the marula fruit fly, *Ceratitis cosyra* (Malod et al. 2017)*,* honey bees (*Apis mellifera*) (Archer et al. 2014; Paoli et al. 2014), and the crickets *Teleogrylls commodus* (Rapkin et al. 2017) and *Gryllus veletis* (Harrison et al. 2014)*.* High protein intake also reduces survival in mice (Solon-Biet et al. 2015) and low intake of animal-derived proteins in middle-aged adults (~ 50–65 year olds) has positive effects on health and survival, but detrimental effects in the over 65 s (Levine et al. 2014).

While low dietary protein intake improved lifespan, it also reduced female egg production. This finding is consistent with previous *Drosophila* research (discussed below) and insect work more broadly, where egg or offspring production is optimised on diets that are more balanced in their P:C ratio. For example, egg production is greatest on a P:C ratio of between 1_P_:1_C_ (Fanson and Taylor 2012) and 1_P_:2.3_C_ in Queensland fruit flies (Fanson et al. 2009), 1_P_:2.5_C_ in marula fruit flies (Malod et al. 2017), 1_P_:1_C_ in the Australian black field cricket (Maklakov et al. 2008; Rapkin et al. 2017) and 3_P_:1_C_ in the field cricket *G. veletis* (Harrison et al. 2014)*.* The positive effects of protein on female reproductive success likely reflects that in many insects, protein helps stimulate oogenesis and regulate vitellogenesis (Wheeler 1996). However, there are exceptions to this general pattern; in the cockroach *Naupheota cinerea* female clutch size improves with carbohydrate consumption and is independent of protein consumed during adulthood (Bunning et al. 2016).

Male fertility does not appear to rely so heavily on protein consumption. In the insects studied to date, male reproductive effort is typically maximised on low protein, high carbohydrate diets (Maklakov et al. 2008; Jensen et al. 2015; Rapkin et al. 2017). Although once more there are exceptions to this general trend; protein consumption increases sexual signaling in the field cricket *G. veletis* (Harrison et al. 2014), courtship activity in male sticklebacks (*Gasterosteus aculeatus*) (Moatt et al. 2019) and testes mass in mice (Solon-Biet et al. 2015). In the current study, males conformed to the more general insect trend, maximizing their lifespan and reproductive output when fed the same low protein, high carbohydrate nutrient blend. Accordingly, we find that females experienced a nutrient-based trade-off between lifespan and daily reproductive investment (different nutrient blends increase expression of each trait) but males did not.

Males might not always manage to avoid a dietary mediated trade-off between survival and reproductive rates. For males to fertilize ova they often need to outcompete their rivals, court females via energetically expensive displays and transfer functional sperm and seminal fluid (Hosken et al. 2019; Archer and Hosken 2021). Each of these reproductive traits may be optimised on different nutritional blends. In cockroaches for example, high carbohydrate intake allows males to invest heavily in pheromones that increase their attractiveness (South et al. 2011), but high protein intake improves sperm numbers (Bunning et al. 2015). This means that the nutrient blend that increases success in pre-copulatory sexual selection differs from the diet that promotes male success in post-copulatory sexual selection. Accordingly whether (and how much) dietary optima diverge between longevity and reproductive effort, depends on the traits in which males must invest to produce offspring (Moatt et al. 2019). This almost certainly differs between species and environments that vary in the intensity of male-male competition before and after mating (Kokko and Rankin 2006). It will remain unclear how often males avoid a lifespan-reproductive trade-off until we better quantify how reproductive effort is affected by diet in both sexes in a broad range of animal taxa.

A secondary aim of our work was to create nutrient landscapes using fully chemically defined, agar-based diets and thus avoid using the CAFE approach, which reduces *D. melanogaster* fitness (Lee et al. 2008; Jensen et al. 2015). Rather than measuring dietary intake, the standard method to create nutrient landscapes (Simpson and Raubenheimer 1995), we created landscapes by mapping phenotype (lifespan, daily reproductive effort and total reproductive effort) to the protein and carbohydrate content of 40 experimental diets to achieve a broad, detailed coverage of the nutrient space. We find that dietary optima for the traits assessed here were in similar regions of the nutrient landscape to previous work using the CAFE method. For example, the dietary optima for lifespan here (1_P_:15.93_C_ in males, 1_P_:15.88_C_ in females) are almost identical to those identified by Jensen et al. (2015) (1_P_:16_C_), despite the two studies using different nutrient ratios and concentrations. The dietary optima identified here are even similar to estimates from studies using different diet formulations (e.g. where yeast is used a protein source), although there is some sex-specific variation between some studies (Lee et al. 2008; Bruce et al. 2013; Kim et al. 2020; Skorupa et al. 2008).

Dietary optima for male reproductive traits observed here are similar to those identified by Jensen et al. (2015) (i.e. daily offspring sired optimised at 1_P_:12.78_C_ versus 1_P_:16_C_). Although both estimates differ from the 1_P_:2_C_ ratio that optimised male reproductive output in work by Reddiex et al. (2013) and the 1_P_:9_C_ estimate from Morimoto & Wigby (2016). These other studies tested how diet affects male reproduction in the days following adult eclosion, while Jensen et al. (2015) and our present study tested these effects over the entire life-course. In fact, the nutrient ratio that maximised offspring production in young male flies here (i.e. 5 days post-eclosion) was ~ 1_P_:2_C_. This suggests that the nutrient blend associated with high male reproductive output in the short term may differ from that generating higher reproductive output over the entire life-course. Once again, understanding nuanced effects of diet on male reproductive capacity relies on collecting more data linking nutrition and reproduction in both sexes, in more species.

Finally, the dietary optima we identify for female daily fecundity (1_P_:1.16_C_) are similar to the 1_P_:2_C_ ratio identified by Jensen et al. (2015), as well as earlier work in *D. melanogaster* using yeast as a protein source (Lee et al. 2008). Our results diverge from previous published estimates when considering total (rather than daily) reproductive success in females, which we find is greatest at a higher protein intake than in previous work. We suspect that this is because particularly pronounced improvements in fecundity in females fed agar based foods (discussed below), drive dietary optima for total reproductive success towards higher protein nutrient blends.

While the nutrient blends that optimise lifespan and reproduction are similar between studies, female fitness was substantially higher in flies fed agar diets. For example, females fed the optimal agar diet for lifespan lived around 11.67 days longer than females fed the optimal diet for lifespan in Jensen et al. (2015). Egg laying rates are also consistently high in the current study. These improvements suggest that agar based diets do not have the costly phenotypic effects in females that are characteristic of the CAFE approach.

While males fed the optimal agar diet for lifespan survived for 8.2 days longer than individuals fed the optimal liquid diet in Jensen et al. (2015), for some equivalent P:C ratios flies lived longer on the CAFE diets than on agar based foods. If flies cannot consume CAFE diets as readily (Moatt et al. 2020), it may be easier to overconsume suboptimal nutrient blends when fed agar diets. This would explain why males fed optimal P:C ratios perform better on agar diets, but these benefits are lost as diets become more imbalanced. While this idea is speculative, it raises the possibility that use of the CAFE approach may have concealed the magnitude of effects of nutritional imbalances on phenotype. More work is needed to test this idea. Further, male fertility was broadly equivalent in the current study and in Jensen et al. (2015). This may reflect the influence of the agar based diets. Overall, it is hard to reconcile consistently positive effects on females of agar based diets with more equivocal impacts on males. Moreover, while we see pronounced improvements in fitness traits in the current work relative to flies fed via the CAFE approach, our fitness trait values are still on the low side—virgin females from the Genetic Reference Panel lines (DGRP) live for 55.28 days (Ivanov et al. 2015), which is around 20 days longer than the average lifespan of flies fed that diets best for survival here. There may be many reasons for this (e.g. our flies were mated and mating can be costly in *D. melanogaster,* we assayed flies on novel experimental diets rather than the foods they have adapted to over generations of lab adaptation etc.), but this suggests that there is further room for diet optimisation. In particular, male fertility values in the current study are low– a possible explanation for this that warrants investigation is cytoplasmic incompatibility (Werren et al. 2008) given that female fecundity (measured as egg counts) was high, while male fertility (measured as offspring sired in competitive assay) was low and the *Wolbachia* status of stocks was not known.

Measuring consumption is vital to testing how individuals self-regulate their dietary intake and to detect compensatory feeding. The CAFE method remains the best way to do this on a large scale (Deshpande et al. 2014). Accordingly, the conclusion that despite costly effects on fitness the CAFE approach does not appear to skew the qualitative relationship between nutrients and phenotype is reassuring. However, we show that it is possible to create an informative, high resolution nutrient landscape without measuring food consumption. When might it be more appropriate to adopt this alternative approach? There are clear scenarios where it is not appropriate to measure consumption using a technique that reduces fitness—for example when characterising how nutrition affects detailed demographic measures of aging. More generally, there may be merit in investing the time saved from measuring dietary intake towards increasing sample sizes for researchers interested in phenotypes that require large sample sizes (e.g. calculating age-dependent mortality rates, characterising behaviour). However, the most obvious case where it makes sense not to measure consumption is in species that live, excrete on and reproduce in their own food. *Drosophila* are an example of this but so are nematodes and numerous pests of stored products (e.g. flour beetles—House et al. 2016). Perhaps creating landscapes without measuring consumption will enable researchers to expand the taxonomic scope of research using the Geometric Framework? Although if individual nutrient intake is not assayed then it is vital that a larger number of experimental diets are used to cover nutrient space with sufficient resolution to produce a meaningful landscape. Ultimately, the advent of tools that allow us to better measure dietary intake of agar-based foods may mean we do not have to choose between the costs and benefits of either approach. In the interim, it is reassuring that the relationship between food and phenotype is strikingly similar in *D. melanogaster*, whichever method researchers choose to use.

## Supplementary Information

Below is the link to the electronic supplementary material.

Supplementary file1 (DOCX 314 kb)

## Data Availability

Data will be archived on manuscript acceptance.

## Code availability (software application or custom code)

NA.
