# Supplementary material for: Mapping sex differences in the effects of protein and carbohydrates on lifespan and reproduction in Drosophila melanogaster: is measuring nutrient intake essential?
Source: Biogerontology. 2022 Feb 5;23(1):129–44. doi: 10.1007/s10522-022-09953-2 (PMC8888493; doi:10.1007/s10522-022-09953-2)
Supplement: Supplementary file 1 — Supplementary file1 (DOCX 314 kb) [file 10522_2022_9953_MOESM1_ESM.docx]

**Supplementary Information**

**Mapping sex differences in the effects of protein and carbohydrates on lifespan and reproduction in *Drosophila melanogaster*: is measuring nutrient intake essential?**

Matthew R. Carey^1,2^, C. Ruth Archer^2,3^, James Rapkin^2^, Meaghan Castledine^2^, Kim Jensen^4^, Clarissa M. House^5^, David J. Hosken^2^, John Hunt^5^

^1.^ Department of Metabolism, Digestion and Reproduction, Imperial College London, London, UK

^2.^ Centre for Ecology and Conservation, University of Exeter, Cornwall Campus, UK

^3.^ Institute of Evolutionary Ecology and Conservation Genomics, University of Ulm, Albert-Einstein Allee 11, 89069 Ulm, Germany

^4.^ Department of Animal Science - ANIS Nutrition, Aarhus University, Tjele, Denmark

^5.^ School of Science, Western Sydney University, Hawkesbury Campus, Richmond, NSW, Australia

**Contents**

Text S1. The sequential model building approach for comparing nutritional landscapes.

Table S1. Experimental Diet Composition.

Table S2. The mean and standard error (in brackets) lifespan (LS), daily reproductive effort (DRE) and total reproductive effort (TRE) in males and females on each of the 40 artificial diets.

Fig S1. The percentage of dietary protein and carbohydrate in experimental diets.

Fig S2. Schematic illustrating female experimental schedule.

Fig S3. Schematic illustrating male experimental schedule.

Fig S4. Theta (***θ***) and the Euclidean distance (***d***) used to determine the degree of difference in the effects of dietary protein and carbohydrate on different traits across and within the sexes.

**Text S1. Sequential model building approach**

We used a sequential model building approach to assess whether the linear and nonlinear effects of dietary protein and carbohydrate differed for our response variables (Draper & John 1988; South et al., 2011). In our analyses, the different response variables were lifespan (LS), daily reproductive effort (DRE) and total reproductive effort (TRE). We compared the effects of macronutrients in experimental diets on the same response variables across the sexes, and on different response variables within each sex. As these responses variables were measured in different units, it was necessary to standardize them to a mean of zero and standard deviation of one using a *Z*- transformation prior to comparison.

To start the sequential model, it was necessary to generate a “dummy variable” for the specific comparison being conducted: when comparing the same response variables across the sexes, the dummy variable would be “sex” but when comparing different response variables within a sex the dummy variable would be response “trait”. These dummy variables were used to code each data point so that the analysis could distinguish between the data sets being compared. The following General Linear Model was then run (in this case, *Sex* is the dummy variable as we are comparing the same response variable across the sexes):

$R=\beta_{0}+\alpha_{0}Sex+\sum_{i=1}^{n} \beta_{i}N_{i}+\varepsilon$ (Eq.1)

where *R* is the standardized response variable, *N_i_* refers to the intake of the *i*th nutrient, *n* represents the number of nutrients contained in the model and *ε* is the unexplained error. This model was fit by including the dummy variable (as a fixed effect), dietary protein and carbohydrates as covariates and the standardized response variable. This is referred to as the reduced model as it only contains the linear effects of dietary protein and carbohydrates on the response variable being compared. By fitting (Eq.1), the unexplained (i.e. residual) sums of squares for this reduced model (*SS_R_*) was extracted. A second (complete) model was then fit that includes all of the terms in (Eq.1), plus the term *α_i_N_i_Sex,* which represents the linear interaction of *Sex* and the *i*th nutrient:

$R=\beta_{0}+\alpha_{0}Sex+\sum_{i=1}^{n} \beta_{i}N_{i}+\sum_{i=1}^{n} \alpha_{i}N_{i}Sex+\varepsilon$ (Eq.2)

The residual sums of squares was then extracted from this complete model (*SS_C_*). A partial *F*-test (Bowerman & O'Connell 1990) was used to compare *SS_R_* and *SS_C_* from (Eq.1) and (Eq.2) respectively:

$F_{a,b}=\frac{\left( {SS}_{R}-{SS}_{C} \right)/a}{\left( {{SS}_{C}}/b \right)}$ (Eq.3)

where *a* is the number of terms that differ between the reduced and complete model (i.e. the number of interaction terms added to (Eq.2) and *b* is the error degrees of freedom for *SS_C_*. A significant *F_a,b_* indicates that the inclusion of the interaction terms in (Eq.2) significantly improved the fit of the model and that the linear effects of dietary protein and carbohydrates on the response variables differ across the sexes. Inspection of the interaction terms for each nutrient from the complete model (Eq.2) can be used to determine which nutrient(s) were responsible for the significance of the overall partial *F*-test.

Next, we tested whether the quadratic effects of dietary nutrients on the response variable differed between the sexes. Again, we built a reduced General Linear Model that included both the linear and quadratic effects of dietary nutrients:

$R=\beta_{0}+\alpha_{0}Sex+\sum_{i=1}^{n} \beta_{i}N_{i}+\sum_{i=1}^{n} \alpha_{0}N_{i}Sex+\sum_{i=1}^{n} \beta_{i}N_{i}^{2}+\varepsilon$ (Eq.4)

Again, we extracted the *SS_R_* from this reduced model. We then ran a complete model that includes the interaction terms between *Sex* and the quadratic effects of dietary nutrients on the response variable (*α_i_N_i_^2^Sex*) and extracted the *SS_C_* for this model:

$R=\beta_{0}+\alpha_{0}Sex+\sum_{i=1}^{n} \beta_{i}N_{i}+\sum_{i=1}^{n} \alpha_{i}N_{i}Sex+\sum_{i=1}^{n} \beta_{i}N_{i}^{2}+\sum_{i=1}^{n} \alpha_{i}N_{i}^{2}Sex+\varepsilon$ (Eq.5)

We used (Eq.3) to determine whether the inclusion of the interaction terms improved the fit of the model and, if so, inspected individual interactions terms from (Eq.5) to determine which nutrient(s) contributed to this effect.

Finally, to test whether correlational effects of dietary nutrients on the response variable differed across the sexes, we extracted the *SS_R_* from the reduced model:

$R=\beta_{0}+\alpha_{0}Sex+\sum_{i=1}^{n} \beta_{i}N_{i}+\sum_{i=1}^{n} \alpha_{i}N_{i}Sex+\sum_{i=1}^{n} \beta_{i}N_{i}^{2}+\sum_{i=1}^{n} a_{i}N_{i}^{2}Sex+\sum_{i=1}^{n} \sum_{j\geq1}^{n} \beta_{ij}N_{i}N_{j}+\varepsilon$ (Eq.6)

We then extracted the *SS_C_* from a complete model that included the interaction terms between *Sex* and the correlational effects of dietary nutrients on the response variable (*α_ij_N_i_N_j_Sex*):

$R=\beta_{0}+\alpha_{0}Sex+\sum_{i=1}^{n} \beta_{i}N_{i}+\sum_{i=1}^{n} \alpha_{i}N_{i}Sex+\sum_{i=1}^{n} \beta_{i}N_{i}^{2}+\sum_{i=1}^{n} \alpha_{i}N_{i}^{2}Sex+\sum_{i=1}^{n} \sum_{j\geq1}^{n} \beta_{ij}N_{i}N_{j}+\sum_{i=1}^{n} \sum_{j\geq1}^{n} \alpha_{ij}N_{i}N_{j}Sex+\varepsilon$ (Eq.7)

Again, (Eq.3) was used to determine whether the inclusion of the interaction terms improved the fit of the model and, if so, the individual interactions terms from (Eq.5) were inspected to determine which nutrient(s) contributed to this effect.

**A worked example: comparing the effects of nutrient intake on lifespan across the sexes.**

This numerical example uses the outputs contained in Table 2 of the main manuscript.

Using (Eq.1) and (Eq.2) we can estimate the *SS_R_* and *SS_C_* for the linear effects of dietary protein and carbohydrates across the sexes as 1356.44 and 1343.05, respectively (Table 2). The error degrees of freedom for *SS_C_* is 1594 and the number of interaction terms added to the complete model (Eq.2) is 2. Using (Eq.3), the F value is calculated as 7.95 and with 2, 1594 degree of freedom this equates to a P value of 0.0004 (Table 2). This indicates that the linear effects of dietary protein and carbohydrates on lifespan differs significantly across the sexes. Inspection of the individual interaction terms in (Eq.2) shows that this overall significant effect is due to significant differences in the effects of both dietary protein (*F*_1,1594_ = 8.73, *P* = 0.003) and dietary carbohydrates (*F*_1,1594_ = 7.74, *P* = 0.005) across the sexes (Table 2).

Using (Eq.4) and (Eq.5) *SS_R_* and *SS_C_* for the quadratic effects of dietary protein and carbohydrates across the sexes as 1169.55 and 1164.35, respectively (Table 2). The error degrees of freedom for *SS_C_* is 1590 and the number of interaction terms added to the complete model (Eq.4) is again 2, giving a F value of 3.55 and an associated P value of 0.03 (using Eq.3). Inspection of the individual interaction terms in (Eq.4) shows that this overall significant effect is due to significant differences in the effects of dietary protein (*F*_1,1590_ = 7.08, *P* = 0.008) but not dietary carbohydrates (*F*_1,1590_ = 0.20, *P* = 0.66) across the sexes (Table 2).

Finally, using (Eq.6) and (Eq.7) *SS_R_* and *SS_C_* for the correlational effects of dietary protein and carbohydrates across the sexes as 1137.26 and 1131.30, respectively (Table 2). The error degrees of freedom for *SS_C_* is 1588 and the number of interaction terms added to the complete model (Eq.4) is 1, giving a F value of 8.37 and an associated P value of 0.004 (using Eq.3). As only 1 term is added, inspection of the individual terms is not needed.

**References:**

Bowerman, BL & O’Connell, RT (1990) *Linear Statistical Models: An Applied Approach*. Duxbury, Belmont, California.

Draper, NR & John, JA (1988) Response-surface designs for quantitative and qualitative variables. Technometrics 30: 423-428.

South, SH, House CM, Moore J, Simpson SJ & Hunt J (2011) Male cockroaches prefer a high carbohydrate diet that makes them more attractive to females: implications for the study of condition dependence. Evolution 65: 1594-1606.

**Table S1. Experimental Diet Composition.** A summary of the ingredients to make 500 g of each experimental diet. Diet = the ID number of each experimental diet. The protein portion of the diet contains casein, peptone and albumen in a 3:1:1 ratio, while the carbohydrate portion contains sucrose and dextrin in a 1:1 ratio. Total nutrition refers to the protein and carbohydrate percentage of each diet, and is calculated as the percentage of the solid ingredients in the diet (i.e. excluding linoleic acid) that is protein or carbohydrate. For more information about diet making please see Simpson & Abisgold 1985 Physiol Entoml 10:443-45 https://doi.org/10.1111/j.1365-3032.1985.tb00066.x.

| Diet | Protein (g) | Carbohydrate (g) | Total nutrition (P+C %) | P:C Ratio | Wesson Salts Mix (g) | Vitamin mix (mg) | Cholesterol (mg) | Vitamin C (mg) | Linoleic acid (mL) | Cellulose (g) |
| --- | --- | --- | --- | --- | --- | --- | --- | --- | --- | --- |
| 1 | 45 | 15 | 12 | 3:1 | 12.5 | 900 | 2750 | 1375 | 2.75 | 422.47 |
| 2 | 135 | 45 | 36 | 3:1 | 12.5 | 900 | 2750 | 1375 | 2.75 | 302.47 |
| 3 | 225 | 75 | 60 | 3:1 | 12.5 | 900 | 2750 | 1375 | 2.75 | 182.47 |
| 4 | 315 | 105 | 84 | 3:1 | 12.5 | 900 | 2750 | 1375 | 2.75 | 62.47 |
| 5 | 40 | 20 | 12 | 2:1 | 12.5 | 900 | 2750 | 1375 | 2.75 | 422.47 |
| 6 | 120 | 60 | 36 | 2:1 | 12.5 | 900 | 2750 | 1375 | 2.75 | 302.47 |
| 7 | 200 | 100 | 60 | 2:1 | 12.5 | 900 | 2750 | 1375 | 2.75 | 182.47 |
| 8 | 280 | 140 | 84 | 2:1 | 12.5 | 900 | 2750 | 1375 | 2.75 | 62.47 |
| 9 | 36 | 24 | 12 | 1.5:1 | 12.5 | 900 | 2750 | 1375 | 2.75 | 422.47 |
| 10 | 108 | 72 | 36 | 1.5:1 | 12.5 | 900 | 2750 | 1375 | 2.75 | 302.47 |
| 11 | 180 | 120 | 60 | 1.5:1 | 12.5 | 900 | 2750 | 1375 | 2.75 | 182.47 |
| 12 | 252 | 168 | 84 | 1.5:1 | 12.5 | 900 | 2750 | 1375 | 2.75 | 62.47 |
| 13 | 30 | 30 | 12 | 1:1 | 12.5 | 900 | 2750 | 1375 | 2.75 | 422.47 |
| 14 | 90 | 90 | 36 | 1:1 | 12.5 | 900 | 2750 | 1375 | 2.75 | 302.47 |
| 15 | 150 | 150 | 60 | 1:1 | 12.5 | 900 | 2750 | 1375 | 2.75 | 182.47 |
| 16 | 210 | 210 | 84 | 1:1 | 12.5 | 900 | 2750 | 1375 | 2.75 | 62.47 |
| 17 | 24 | 36 | 12 | 1:1.5 | 12.5 | 900 | 2750 | 1375 | 2.75 | 422.47 |
| 18 | 72 | 108 | 36 | 1:1.5 | 12.5 | 900 | 2750 | 1375 | 2.75 | 302.47 |
| 19 | 120 | 180 | 60 | 1:1.5 | 12.5 | 900 | 2750 | 1375 | 2.75 | 182.47 |
| 20 | 168 | 252 | 84 | 1:1.5 | 12.5 | 900 | 2750 | 1375 | 2.75 | 62.47 |
| 21 | 20 | 40 | 12 | 1:2 | 12.5 | 900 | 2750 | 1375 | 2.75 | 422.47 |
| 22 | 60 | 120 | 36 | 1:2 | 12.5 | 900 | 2750 | 1375 | 2.75 | 302.47 |
| 23 | 100 | 200 | 60 | 1:2 | 12.5 | 900 | 2750 | 1375 | 2.75 | 182.47 |
| 24 | 140 | 280 | 84 | 1:2 | 12.5 | 900 | 2750 | 1375 | 2.75 | 62.47 |
| 25 | 15 | 45 | 12 | 1:3 | 12.5 | 900 | 2750 | 1375 | 2.75 | 422.47 |
| 26 | 45 | 135 | 36 | 1:3 | 12.5 | 900 | 2750 | 1375 | 2.75 | 302.47 |
| 27 | 75 | 225 | 60 | 1:3 | 12.5 | 900 | 2750 | 1375 | 2.75 | 182.47 |
| 28 | 105 | 315 | 84 | 1:3 | 12.5 | 900 | 2750 | 1375 | 2.75 | 62.47 |
| 29 | 10 | 50 | 12 | 1:5 | 12.5 | 900 | 2750 | 1375 | 2.75 | 422.47 |
| 30 | 30 | 150 | 36 | 1:5 | 12.5 | 900 | 2750 | 1375 | 2.75 | 302.47 |
| 31 | 50 | 250 | 60 | 1:5 | 12.5 | 900 | 2750 | 1375 | 2.75 | 182.47 |
| 32 | 70 | 350 | 84 | 1:5 | 12.5 | 900 | 2750 | 1375 | 2.75 | 62.47 |
| 33 | 6.65 | 53.3 | 11.99 | 1:8 | 12.5 | 900 | 2750 | 1375 | 2.75 | 422.52 |
| 34 | 20 | 160 | 36 | 1:8 | 12.5 | 900 | 2750 | 1375 | 2.75 | 302.47 |
| 35 | 33.3 | 266.65 | 59.99 | 1:8 | 12.5 | 900 | 2750 | 1375 | 2.75 | 182.52 |
| 36 | 46.65 | 373.3 | 83.99 | 1:8 | 12.5 | 900 | 2750 | 1375 | 2.75 | 62.52 |
| 37 | 3.5295 | 56.47 | 11.99 | 1:16 | 12.5 | 900 | 2750 | 1375 | 2.75 | 422.47 |
| 38 | 10.59 | 169.41 | 36 | 1:16 | 12.5 | 900 | 2750 | 1375 | 2.75 | 302.47 |
| 39 | 17.645 | 282.355 | 60 | 1:16 | 12.5 | 900 | 2750 | 1375 | 2.75 | 182.47 |
| 40 | 24.705 | 395.295 | 84 | 1:16 | 12.5 | 900 | 2750 | 1375 | 2.75 | 62.47 |

**Table S2. The mean and standard error (in brackets) lifespan (LS), daily reproductive effort (DRE) and total reproductive effort (TRE) in males and females on each of the 40 artificial diets.**

|  | **Female** | | |  | **Male** | | |
| --- | --- | --- | --- | --- | --- | --- | --- |
| **Diet** | LS | DRE | TRE |  | LS | DRE | TRE |
| **1** | 11.10 (1.10) | 19.57 (3.25) | 30.40 (5.47) |  | 8.10 (0.54) | 5.25 (1.49) | 5.25 (1.49) |
| **2** | 14.10 (1.48) | 26.93 (3.22) | 63.30 (8.50) |  | 10.35 (0.81) | 6.35 (1.43) | 7.70 (1.56) |
| **3** | 19.25 (1.79) | 23.84 (2.42) | 81.95 (9.93) |  | 12.95 (1.03) | 8.67 (2.07) | 15.10 (4.25) |
| **4** | 18.85 (1.93) | 28.44 (2.58) | 86.35 (12.18) |  | 14.60 (1.01) | 6.40 (1.02) | 14.10 (2.41) |
| **5** | 11.00 (1.09) | 21.81 (2.61) | 38.30 (6.51) |  | 8.95 (0.46) | 8.95 (2.20) | 10.80 (2.83) |
| **6** | 14.40 (1.30) | 50.85 (2.85) | 104.75 (10.85) |  | 11.25 (0.60) | 7.65 (1.70) | 13.95 (3.74) |
| **7** | 19.80 (2.61) | 41.17 (4.44) | 133.15 (20.98) |  | 15.40 (1.03) | 7.63 (1.46) | 20.50 (4.28) |
| **8** | 18.35 (1.34) | 36.42 (3.23) | 115.60 (13.19) |  | 12.90 (0.72) | 6.83 (1.20) | 13.65 (2.96) |
| **9** | 14.70 (1.69) | 17.91 (2.64) | 49.95 (9.77) |  | 9.70 (0.58) | 5.60 (1.31) | 6.25 (1.63) |
| **10** | 20.85 (1.74) | 40.08 (3.91) | 134.40 (14.75) |  | 13.40 (0.97) | 11.05 (2.13) | 23.70 (5.79) |
| **11** | 23.30 (2.52) | 41.99 (4.83) | 175.25 (22.84) |  | 14.70 (0.97) | 5.17 (0.85) | 12.75 (1.99) |
| **12** | 21.70 (1.67) | 38.10 (1.79) | 144.90 (14.43) |  | 13.40 (0.81) | 5.75 (1.09) | 11.95 (2.43) |
| **13** | 14.10 (2.04) | 19.08 (3.58) | 43.65 (9.57) |  | 10.35 (0.59) | 7.98 (1.79) | 11.25 (2.44) |
| **14** | 20.30 (2.04) | 45.80 (3.35) | 141.95 (14.57) |  | 16.30 (1.28) | 7.13 (1.79) | 17.35 (3.61) |
| **15** | 20.95 (1.81) | 46.42 (3.14) | 158.65 (14.11) |  | 14.25 (1.24) | 4.28 (1.10) | 10.10 (2.59) |
| **16** | 18.95 (1.73) | 53.74 (3.40) | 159.55 (14.65) |  | 13.45 (1.06) | 6.69 (1.38) | 14.95 (3.24) |
| **17** | 18.25 (2.11) | 26.42 (3.83) | 69.76 (9.29) |  | 12.45 (0.69) | 6.88 (1.41) | 13.45 (2.87) |
| **18** | 19.30 (2.27) | 41.06 (4.42) | 129.00 (16.91) |  | 14.50 (0.97) | 11.29 (2.00) | 23.90 (4.15) |
| **19** | 20.45 (2.11) | 35.50 (3.44) | 125.95 (18.03) |  | 11.60 (0.97) | 7.25 (1.76) | 13.90 (3.74) |
| **20** | 19.30 (2.38) | 51.62 (3.43) | 151.60 (17.90) |  | 15.35 (1.42) | 8.65 (1.51) | 21.05 (3.83) |
| **21** | 17.30 (1.86) | 32.10 (2.93) | 74.80 (7.60) |  | 13.10 (1.00) | 8.08 (1.38) | 17.10 (3.39) |
| **22** | 21.70 (2.21) | 44.47 (4.14) | 148.80 (17.37) |  | 17.50 (1.34) | 7.92 (1.76) | 22.10 (4.73) |
| **23** | 24.00 (1.64) | 42.58 (2.41) | 179.50 (14.08) |  | 14.60 (1.13) | 11.00 (2.20) | 24.70 (4.87) |
| **24** | 21.05 (1.85) | 40.40 (2.07) | 140.55 (13.11) |  | 13.75 (0.76) | 7.06 (2.18) | 13.70 (4.20) |
| **25** | 24.85 (2.16) | 21.37 (1.99) | 98.00 (13.00) |  | 17.50 (1.20) | 11.30 (1.49) | 35.10 (5.80) |
| **26** | 26.40 (2.02) | 40.14 (3.31) | 176.90 (18.51) |  | 20.35 (1.69) | 8.99 (1.44) | 33.25 (7.16) |
| **27** | 21.55 (2.43) | 35.56 (3.50) | 141.15 (19.66) |  | 18.00 (1.38) | 9.29 (1.62) | 27.25 (4.53) |
| **28** | 23.50 (1.50) | 32.95 (2.01) | 134.95 (12.77) |  | 17.50 (1.60) | 8.25 (2.04) | 23.45 (5.01) |
| **29** | 27.00 (2.11) | 20.26 (2.34) | 99.35 (12.35) |  | 18.70 (1.33) | 5.94 (0.83) | 19.30 (3.04) |
| **30** | 27.40 (2.17) | 34.65 (2.31) | 158.75 (8.18) |  | 19.80 (1.96) | 12.48 (1.44) | 46.50 (8.18) |
| **31** | 21.65 (2.06) | 32.06 (2.82) | 113.35 (13.22) |  | 18.10 (1.49) | 6.19 (1.16) | 23.85 (5.57) |
| **32** | 26.80 (2.54) | 31.60 (3.36) | 143.35 (16.14) |  | 20.65 (1.69) | 13.71 (1.79) | 43.55 (4.16) |
| **33** | 23.85 (2.20) | 20.99 (2.35) | 83.70 (11.55) |  | 19.75 (1.49) | 9.66 (1.80) | 34.50 (7.14) |
| **34** | 26.30 (2.38) | 33.93 (4.45) | 137.80 (14.63) |  | 28.95 (1.75) | 12.95 (1.25) | 69.05 (7.32) |
| **35** | 25.05 (1.94) | 34.54 (3.23) | 155.35 (14.41) |  | 22.65 (1.82) | 8.99 (1.28) | 36.90 (5.95) |
| **36** | 24.05 (2.45) | 34.13 (2.67) | 136.20 (14.91) |  | 22.10 (1.49) | 10.43 (1.43) | 41.55 (6.18) |
| **37** | 29.65 (1.96) | 15.93 (1.46) | 85.65 (9.03) |  | 20.90 (0.69) | 5.93 (0.90) | 25.80 (3.81) |
| **38** | 36.80 (2.65) | 22.37 (2.14) | 148.10 (14.32) |  | 34.35 (2.22) | 12.57 (1.97) | 70.55 (9.58) |
| **39** | 32.45 (1.81) | 26.43 (2.20) | 149.45 (11.09) |  | 30.95 (2.12) | 17.87 (2.96) | 70.65 (8.08) |
| **40** | 26.15 (2.15) | 26.29 (3.30) | 118.58 (14.58) |  | 25.45 (2.07) | 10.71 (1.04) | 44.86 (5.14) |

**Fig S1. The percentage of dietary protein and carbohydrate in experimental diets.** Each filled circle represents the location of an individual diet in nutritional space. Diets connected by the same dashed line (i.e. nutrient rails) contain equal ratios of protein to carbohydrate (P:C) – each nutrient rail is labelled with its P:C ratio. Diets aligned along the diagonal contain the same total nutrition (i.e. P+C) and the same amount of indigestible carbohydrate i.e. cellulose (e.g. diets with 80% P+C contain 20% indigestible carbohydrate). The shaded polygon represents the location of diets used in Jensen et al. (2015) presented in g / 100 g of liquid diet.


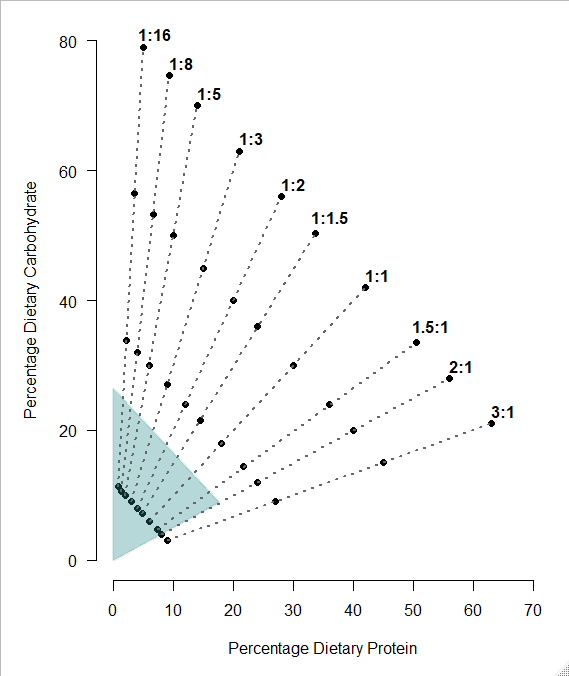


**Fig S2. Schematic illustrating female experimental schedule.** The routine outlined below was used to assay female fecundity, and this schedule was repeated every 5 days across each female’s lifetime. The sum of these counts was used as our measure of **total reproductive effort,** while the average of each individual count was used as our measure of **daily reproductive effort.**


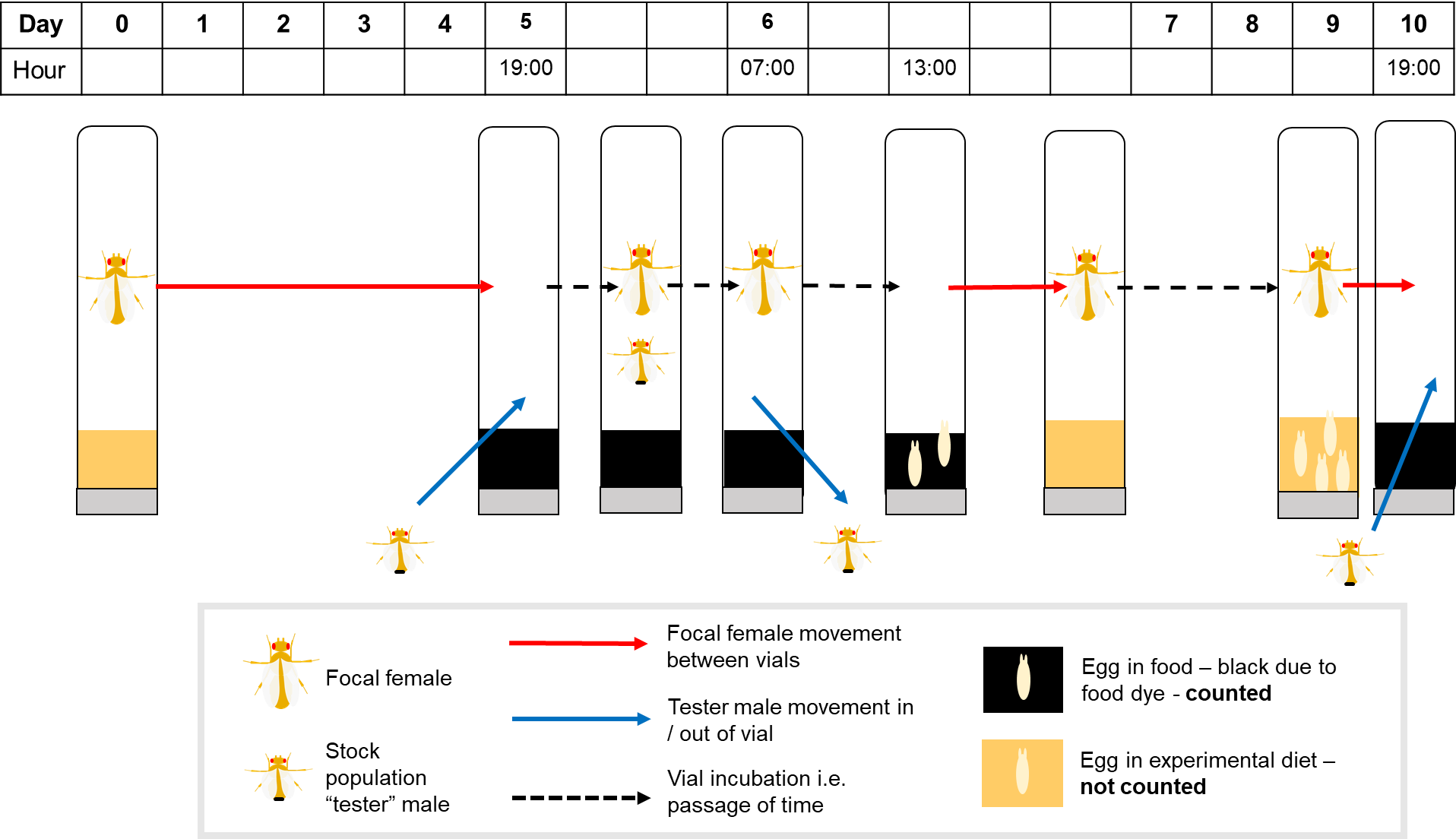


**Fig S3. Schematic illustrating male experimental schedule.** The routine outlined below was used to assay male reproductive success in a competitive assay (i.e. experimental male paired with 1 tester female and 1 mutant competing male). This schedule was repeated every 5 days across each female’s lifetime. The sum of these counts was used as our measure of **total reproductive effort,** while the average of each individual count was used as our measure of **daily reproductive effort.**

**
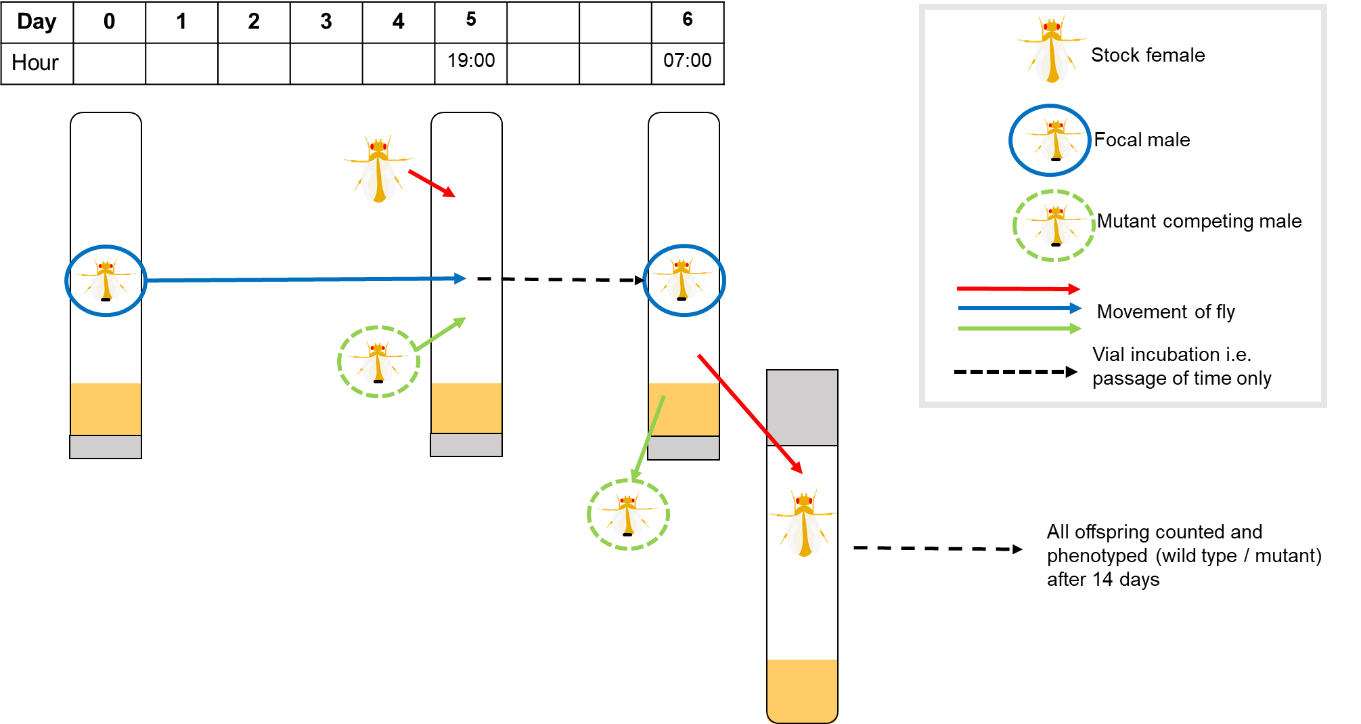
**

**Fig. S4.** An example illustrating theta (***θ***) and the Euclidean distance (***d***) used to determine the degree of difference in the effects of dietary protein and carbohydrate on different traits across and within the sexes. In each panel, the nutritional landscape for two different traits is presented where the darker shading represents an increased expression of the trait and lighter shading represents a decreased expression of the traits. In our study, the two peaks could be for either the same traits (e.g. lifespan, daily or total reproductive effort) in males and females or different traits within the sexes. The black dashed line represents the P:C nutritional rail that passes through the nutritional optima for each trait. The pair of curved, solid black lines that connect the nutritional rails passing through the optima represents the angle (***θ***) between these rails, and the red dashed line represents the Euclidean distance (***d***) between the nutritional optima for each trait. Panels A and B represent the case where the nutritional optima for both traits occur at very similar total nutrient (and caloric) intakes. Thus ***θ*** and ***d*** both adequately capture the difference between the nutritional landscapes. Panel C represents the case where the nutritional optima occur at different total nutrient (and caloric) intakes. This, ***θ*** can be small but ***d*** is large. This highlights the need to use both ***θ*** and ***d*** to quantify the differences between nutritional landscapes.
